# Phylogenetic Analysis of *R2R3-MYB* Family Genes in *Tetrastigma hemsleyanum* Diels et Gilg and Roles of *ThMYB4* and *ThMYB7* in Flavonoid Biosynthesis

**DOI:** 10.3390/biom13030531

**Published:** 2023-03-15

**Authors:** Haishun Xu, Xue Dai, Xue Hu, Haizheng Yu, Ying Wang, Bingsong Zheng, Juan Xu, Xueqian Wu

**Affiliations:** 1 State Key Laboratory of Subtropical Silviculture, Zhejiang A&F University, Hangzhou 311300, China; 2 Zhejiang Provincial Key Laboratory of Characteristic Traditional Chinese Medicine Resources Protection and Innovative Utilization, Zhejiang A&F University, Hangzhou 311300, China

**Keywords:** *Tetrastigma hemsleyanum* Diels et Gilg, *R2R3-MYB*, flavonoid biosynthesis, hairy root

## Abstract

*Tetrastigma hemsleyanum* Diels et Gilg (*T. hemsleyanum*) is an extensively used Chinese folk herb with multiple bioactivities. Among these bioactivities, flavonoids are recognized as the representative active ingredients. We previously found an elevated accumulation of flavonoids in *T. hemsleyanum* under water stress; however, the mechanism remains unclear. R2R3-MYB transcription factors play vital roles in the plant response to environmental stress and the regulation of secondary metabolites. Herein, a systematic transcriptome identification of *R2R3-MYB* family genes under water stress in *T. hemsleyanum* was performed to explore their potential function in the biosynthesis of flavonoids. A total of 26 *R2R3-MYB* genes were identified, most of which were clustered into functional branches of abiotic stress. *ThMYB4* and *ThMYB7* were then screened out to be associated with the biosynthesis of flavonoids through a protein-protein interaction prediction. An expression correlation analysis based on RNA-seq further confirmed that *ThMYB4* and *ThMYB7* were positively related to the flavonoid biosynthetic pathway genes of *T. hemsleyanum*. In *ThMYB4*- and *ThMYB7*-overexpression hairy roots, it was found that the expression of *ThCHS* and *ThCHI* was significantly increased, suggesting that *ThMYB4* and *ThMYB7* may act as regulators in flavonoid biosynthesis. This will shed new light on the promotion of flavonoid production and the medicinal value of *T. hemsleyanum* by manipulating transcription factors.

## 1. Introduction

*Tetrastigma hemsleyanum* Diels et Gilg (*T. hemsleyanum*), which belongs to the grape family Vitaceae, is an extensively used Chinese folk herb with multiple bioactivities. It has been reported to exhibit anti-inflammatory, analgesic, antipyretic, antitumor, and antiviral properties [1,2,3]. Flavonoids are recognized as the representative active ingredients in *T. hemsleyanum*, and more than 30 flavonoids have been isolated and identified [4]. As an extremely important class of bioactive secondary metabolites in *T. hemsleyanum*, flavonoids have problems with respect to their low content and unstable quality and cannot satisfy the demands of the market [2]. Meanwhile, the mechanisms of flavonoid biosynthesis and metabolic regulation are unclear. Flavonoids are generated from the phenylalanine through phenylpropanoid pathways. The genes code for the enzymes including phenylalanine ammonia-lyase (PAL), cinnamate-4-hydroxylase (C4H), 4-coumarate-CoA ligase (4CL), chalcone synthase (CHS), chalcone isomerase (CHI), flavanone 3-hydroxylase (F3H), dihydroflavonol reductase (DFR), and flavonol synthase (FLS) that involved in this pathway [5].The accumulation of flavonoids often occurs in plants subjected to abiotic stresses so as to resist environmental stresses such as water, soil salinity, and extreme temperatures [6,7,8]. A large number of studies indicated that the biosynthesis of flavonoids was affected by water stress. For instance, a water deficit affected the accumulation of flavonoids in *Scutellaria baicalensis* [9], and water stress regulated the biosynthesis of flavonoids in *grapes* [10]. We previously found that the content of flavonoids increased under water stress in *T. hemsleyanum*; however, the molecular mechanism of flavonoid regulation remains unknown.

During their long history of survival, plants have developed defense mechanisms to resist abiotic stress, and the transcription factors involved in transcriptional regulation are presumed to be the key among many factors [11,12]. Transcription factor (TF), a stress-response regulator [13], is indispensable for the metabolic regulation of flavonoid biosynthesis [14]. MYB (v-myb avian myeloblastosis viral oncogene homolog), as one of the largest plant-specific TF families, contains highly conserved MYB domains which have approximately 52 amino acids with three α-helices in each repeat [15]. According to the number of repeat sequences, MYB transcription factors are divided into four subfamilies, including R1-MYB, R2R3-MYB, 3R-MYB, and 4R-MYB factors [15,16], respectively, and are extensively represented in all eukaryotic forms. The first plant MYB gene was isolated from maize, which encodes a c-MYB-like [17]. Since then, plant MYB genes have been discovered and studied. It has been confirmed that R1-MYB mainly participates in cell and organ morphogenesis [18], 3R-MYB primarily functions in the cell cycle [19], and 4R-MYB is rarely reported [20]. The most noteworthy subgroup is the *R2R3-MYB* family, which has great significance in the process of anti-stress physiology [21,22,23]. The *AtMYB52* was shown to confer ABA hypersensitivity and drought tolerance in *Arabidopsis thaliana* [24], and R2R3-MYB members *TaMYB31* [25], *OsMYB6* [26], and *BpMYB123* [27] from *Triticum aestivum*, *Oryza sativa*, *and Betula platyphylla*, respectively, were also proven to improve drought tolerance. It has also been indicated that the R2R3-MYB subfamily is closely related to the accumulation of flavonoids in plants under water stress. For instance, *AtMYB4* [28], *NtMYB3* [29], *CsMYB2*, *CsMYB26* [30], *CsMYB33*, and *CsMYB78* [31] were shown to regulate flavonoid biosynthesis in *Arabidopsis thaliana*, *Narcissus*, tea, and *Cannabis sativa.* These findings indicate that R2R3-MYB transcription factors take part in the flavonoid biosynthesis. Hence, it is of great importance to reveal the R2R3-MYB transcription factors related to flavonoid biosynthesis that respond to water stress in *T. hemsleyanum*.

In this study, a systematic transcriptome identification of the *R2R3-MYB* family genes and a protein–protein interaction prediction were performed to identify the *R2R3-MYB* transcription factors related to flavonoid biosynthesis in *T. hemsleyanum*. Moreover, transgenic *T. hemsleyanum* hairy roots were constructed to verify the regulatory effects of *R2R3-MYB* on flavonoid metabolism. This will shed new light on the promotion of flavonoid production and the medicinal value of *T. hemsleyanum* by manipulating transcription factors.

## 2. Materials and Methods

### 2.1. Plant Materials and Treatments

The transcriptome sequence data used in this study were previously generated and validated in our laboratory. *T. hemsleyanum* seedlings were collected from the Sanyeqing Base in Suichang County, Zhejiang Province, China. The water treatments were as follows: a 34% water content was taken as the soil saturated water content, and different water treatments were set according to the soil relative water content. A total of 75 *T. hemsleyanum* seedlings were randomly divided into three groups: the control group (70~80% relative water content), the drought group (20~30% relative water content), and the waterlogging group (90~100% relative water content). The transcriptome data of *T. hemsleyanum* were simultaneously obtained from the roots and leaves under different water treatments. Each treatment contained three biological replicates.

### 2.2. Transcriptome-Wide Identification of the ThR2R3-MYB Proteins

The original sequences of the MYB in *T. hemsleyanum* were downloaded from the transcriptome data. The ThR2R3-MYB proteins with two MYB conserved domains were identified using NCBI (https://www.1ncbi.nlm.nih.gov/Structure/bwrpsb/bwrpsb.cgi) and Smart BLAST (https://blast.ncbi.nlm.nih.gov/smartblast/?LINK_LOC=BlastHomeLinkto) that accessed on 5 February 2022 Ultimately, all the R2R3-MYB proteins were manually inspected using ClustalX to further ensure that the gene models contained the two MYB domains [32].

### 2.3. Physicochemical Properties and Subcellular Location of R2R3-MYB

The physicochemical properties of the ThR2R3-MYB proteins, including the relative molecular weight, isoelectric point, instability coefficient, and total average hydrophilicity, were analyzed using the ExPASy online tool [33] (https://web.expasy.org/protparam/) that accessed on 10 March 2022. To master more biological functions of the R2R3-MYB proteins, the WoLF PSORT [34] (https://wolfpsort.hgc.jp/) that accessed on 10 March 2022 was used to predict the subcellular location of the R2R3-MYB proteins.

### 2.4. Conserved Motifs of R2R3-MYB Proteins

The conserved motifs of each R2R3-MYB protein were defined by the MEME program [35] (http://meme-suite.org/) that accessed on 12 March 2022 with the motif number set at 20 and the other parameters as default.

### 2.5. Phylogenetic Analysis of R2R3-MYB Gene Family

The R2R3-MYB proteins from *Arabidopsis* and *Vitis vinifera*, obtained from TAIR (https://www.Arabidopsis.org/) and PLEXdb (http://www.plexdb.org/) that accessed on 15 March 2022, respectively, were set as the query sequences. Then, all R2R3-MYB protein sequences from *T. hemsleyanum*, *A. thaliana*, and *V. vinifera*, were aligned using ClustalW. The phylogenetic analysis was conducted in the MEGA11 program using the neighbor-joining (N-J) method, with a bootstrap analysis of 1000 replicates and a P-distance model [36]. Finally, the phylogenetic tree was decorated on EVOLVIEW online (https://www.evolgenius.info/evolview-v2/#login) that accessed on 16 March 2022. The classification of the R2R3-MYB proteins was performed according to their phylogenetic relationships with the *A. thaliana* and *V. vinifera* R2R3-MYB proteins.

### 2.6. Expression Patterns of ThR2R3-MYB Genes

The expression levels of 26 *ThR2R3-MYB* genes were calculated as transcripts per kilobase of exon model per million mapped reads (TPM) units from the transcriptome analysis. Heat maps were generated, and hierarchical clustering was performed using TBtools v1.108 software.

### 2.7. Protein Interaction Network and Tertiary Structure Prediction

Interaction networks of the R2R3-MYB proteins were predicted using the online site STRING [37] (https://string-db.org/) that accessed on 27 March 2022. *Arabidopsis* were used as reference species. R2R3-MYB proteins were shown in the red node with *Arabidopsis* orthologs. The sequences were uploaded to the SWISS-MODEL [38] (https://swissmodel.expasy.org/interactive) that accessed on 29 March 2022, which provided a 3D protein model prediction service. The models were then decorated by VMD.

### 2.8. Subcellular Localization and Transcriptional Activity of the Candidate Genes

To further explore the characterization of the two candidate genes, *ThMYB4* and *ThMYB7*, the localization and transcriptional activity were researched. Firstly, the CDS sequences of *ThMYB4* and *ThMYB7* were fused into the vector *A. tumefaciens* GV3101 (WEIDI, Shanghai, China) with GFP. They were then transformed into tobacco leaves. Finally, fluorescence images were collected using a laser confocal microscope (FV3000, Olympus, Japan) to visualize the subcellular localization. To reveal the transcriptional activity, we integrated the CDS sequences into the vector pGBKT7 and then transformed the fusion vector into an AH109 competent cell (WEIDI, Shanghai, China), which was subsequently cultured on SD/-Trp (TaKaRa, Kyoto, Japan) and SD/-Trp-His-Ade (TaKaRa, Kyoto, Japan) selective media with X-α-gal for 48–72 h at 28 °C.

### 2.9. Expression Profiles of the Genes of Flavonoid Biosynthesis in T. hemsleyanum

To analyze the expression patterns of the genes related to flavonoid biosynthesis under water stress, we obtained TPM values to generate a heatmap via the TBtools v1.108 software. The TPM average values were used to normalize the expression level of the genes in the flavonoid biosynthesis of *T. hemsleyanum* under drought and waterlogging stress, respectively.

### 2.10. Correlation Analysis of the R2R3-MYB Genes and Flavonoid-Biosynthesis Enzyme Genes

The correlation coefficients between the two candidate genes and the flavonoid biosynthesis enzyme genes were normalized to TPM by using the R package. The red colors represented positive correlation coefficients, and the blue colors represented negative correlation coefficients. The correlation coefficients were between positive one (+1) and negative one (−1).

### 2.11. Construction of Plant Expression Vectors

To investigate the regulatory effect of *ThMYB4* and *ThMYB7* on flavonoid biosynthesis, transgenic *T. hemsleyanum* hairy roots that overexpressed *ThMYB4* and *ThMYB7* were constructed. For the construction of the *ThMYB4*- and *ThMYB7*-overexpressed vectors, the CDS sequences of *ThMYB4* and *ThMYB7* were cloned and inserted into the overexpression vector pK7WG2R at the restriction site between BamHI and SalI (NEB, Beijing, China) to generate pK7WG2R-ThMYB4 and pK7WG2R-ThMYB7.

### 2.12. Acquisition of Transgenic Hairy Root Lines

The hairy root lines were acquired from the transformation of leaf explants from the sterile plantlets of *T. hemsleyanum*. To identify the positive transgenic lines, all hairy roots were collected to isolate the genomic DNA using the Super Plant Genomic DNA Kit (TIANGEN, Beijing, China). The positive transgenic lines were then identified by PCR using *ThMYB4* and *ThMYB7* specific primers, respectively. The identified positive lines were used for the extraction of RNA and metabolites. All primers used for the construction of the overexpression vector and the PCR identification of transgenic lines are listed in Table S1.

### 2.13. Quantitative Real-Time PCR

The primers of the flavonoid pathway genes *ThPAL*, *ThC4H*, *Th4CL*, and *ThCHS* in *T. hemsleyanum* were designed using the Primer Premier 6.0. The actin gene was used as an internal reference to normalize the data. All primers are listed in Table S1.

The RNAprep Pure Plant Plus Kit Tissue Kit (TIANGEN, Beijing, China) was used to isolate the total RNA from positive transgenic hairy root lines. The total RNA of 500 ng was used to perform a reverse transcription analysis using the PrimeScript^TM^RT Master MIX (TaKaRa, Kyoto, Japan). The amplification reaction was performed in the Applied Biosystem Real-Time PCR System with an optional 96-well plate. The amplification profile was 95 °C for 30 s, followed by 40 cycles of 95 °C for 5 s and 60 °C for 30 s. Three biological and technical replicates were used for each assay. The relative gene expression was calculated by the relative quantitative analysis method (2^−∆∆Ct^). Statistical significance was determined using the one sample *t*-test and a one-way analysis of variance using SPSS Statistics 26.

## 3. Results

### 3.1. Physicochemical Property and Subcellular Location of R2R3-MYB in T. hemsleyanum

A total of 26 *T. hemsleyanum* R2R3-MYB members were identified, according to the transcriptome analysis. In order to grasp the basic characteristics of the R2R3-MYB proteins, we predicted their physicochemical properties and localization information. As listed in Table 1, the molecular weights ranged from 13.22 kDa (*ThMYB6*) to 85.90 kDa (*ThMYB1*), and the theoretical pI values ranged from 5.19 (*ThMYB15*) to 10.56 (*ThMYB18*). The protein instability indices were all more than 40, indicating that these R2R3-MYB proteins were unstable. The Aliphatic indices were mainly distributed between 60 and 80, demonstrating convincingly that the aliphatic amino acids occupied a higher proportion. The predicted GRAVY scores of the R2R3-MYB proteins anged from −1.223 (*ThMYB16*) to −0.430 (*ThMYB23*), suggesting that all the R2R3-MYB proteins were hydrophilic in nature. The subcellular localization prediction showed that 21 out of the 23 (91.30%) R2R3-MYB proteins might locate in the nucleus.

### 3.2. Conserved Motifs in R2R3-MYB Proteins

**Figure 1:**
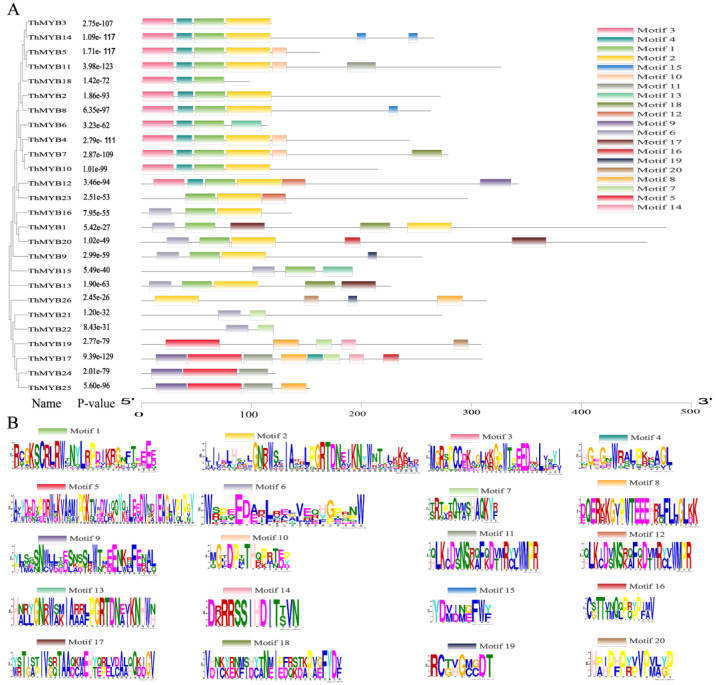
Conserved motifs in R2R3-MYB proteins. (**A**) Multiple colored boxes represent different motifs. (**B**) The motif sequence information.

A neighbor-joining phylogenetic tree of 26 R2R3-MYB proteins was constructed. The MEME program was used to search for conserved motifs in the R2R3-MYB proteins to display the diversification of R2R3-MYB in *T. hemsleyanum*. In total, 20 conserved motifs were identified and designated as motifs 1 to 20. The motif sequence information was provided (Figure 1B). As shown in Figure 1A, almost all R2R3-MYB family members contained four highly conserved motifs, 1, 2, 3, and 4, which represent the R2 and R3 MYB domains [15]. Aside from the highly conserved motifs composed of MYB domains, the members of the same subgroup were found to exhibit other similar motif compositions. For example, motif 5 existed in ThMYB19, ThMYB17, ThMYB24 and ThMYB25, and motif Six existed in ThMYB16, ThMYB1, ThMYB20, ThMYB9, ThMYB15, ThMYB13, ThMYB21, and ThMYB22, suggesting functional similarities within the same subclade. In general, according to similar conservation domains, the member of subfamilies was classified in the phylogenetic tree. The accuracy and authenticity of the classification in the phylogenetic tree were verified by these characteristics.

### 3.3. Phylogenetic Relationship and Evolutionary Analysis of R2R3-MYB TFs

**Figure 2:**
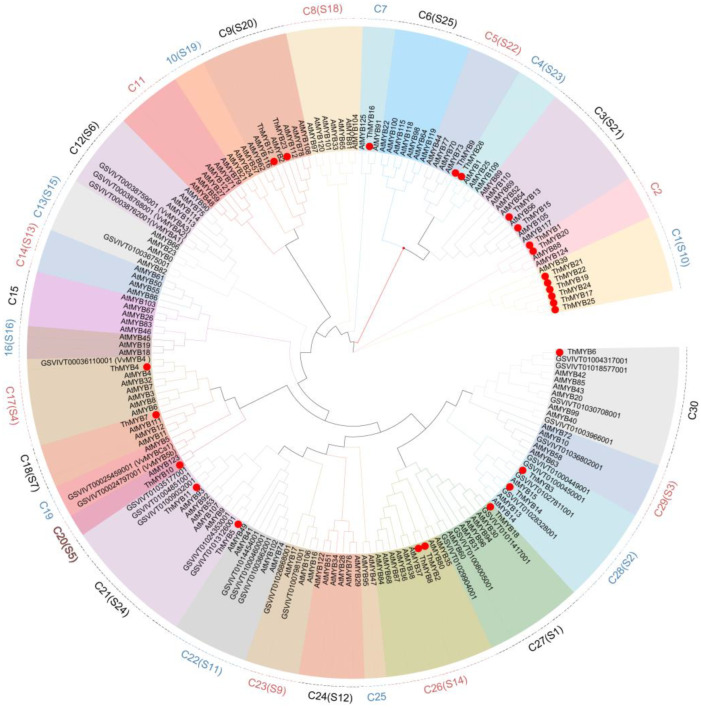
Phylogenetic tree of R2R3-MYB transcription factors (TFs) in *T. hemsleyanum*, *A. thaliana*, and *V. vinifera*. The neighbor-joining (NJ) phylogenetic tree was constructed using the MEGA11 program with 1000 bootstrap replicates, and the R2R3-MYB proteins are labeled by circles with red dots.

To study the evolutionary relationship among the R2R3-MYB TFs in *T. hemsleyanum*, an unrooted neighbor-joining (NJ) phylogenetic tree was constructed using MEGA11.0 to align the identified R2R3-MYB sequences with *A. thaliana* and *V. vinifera*. Based on the classification proposed by Doubs et al. [15], the evolutionary tree was subdivided into 30 clades for the R2R3-MYB subfamilies, designated C1C30 (Figure 2). According to the sequence similarity and topology, all R2R3-MYBs in *T. hemsleyanum* were clustered into 15 clades, manifesting that the *T. hemsleyanum* R2R3-MYB members were closely related to the *A. thaliana* and *V. vinifera* homologous proteins in sequence and structure. Studies have shown that subgroups (C17(S4) and C18(S7)) were involved in the branches of defense responses and flavonoid biosynthesis regulation [39]. The *ThMYB4* and *ThMYB7* grouped into 17(S4) and C18(S7), respectively, were deduced to be candidate genes for the regulation of flavonoid biosynthesis under abiotic stress in *T. hemsleyanum*. Although R2R3-MYB transcription factors with a high sequence homology in different plants can regulate flavonoid metabolism, they may exhibit different regulatory effects and target different genes for flavonoid biosynthesis. For example, *AtMYB4* and *McMYB4* have sequence similarity and are both capable of regulating flavonoid metabolism; however, *AtMYB4* inhibits the flavonoid accumulation in *Arabidopsis* by suppressing the PAL gene transcription [28], while *McMYB4* promotes flavonoid biosynthesis in apples via regulating CHS expression [40]. Whether *ThMYB4* and *ThMYB7* can regulate the flavonoid metabolism in *T. hemsleyanum* and which flavonoid biosynthetic genes are regulated by them remain unclear. It is of significant importance to verify their regulatory effects on flavonoid regulation.

### 3.4. Expression Profiles of ThR2R3-MYB Genes

**Figure 3:**
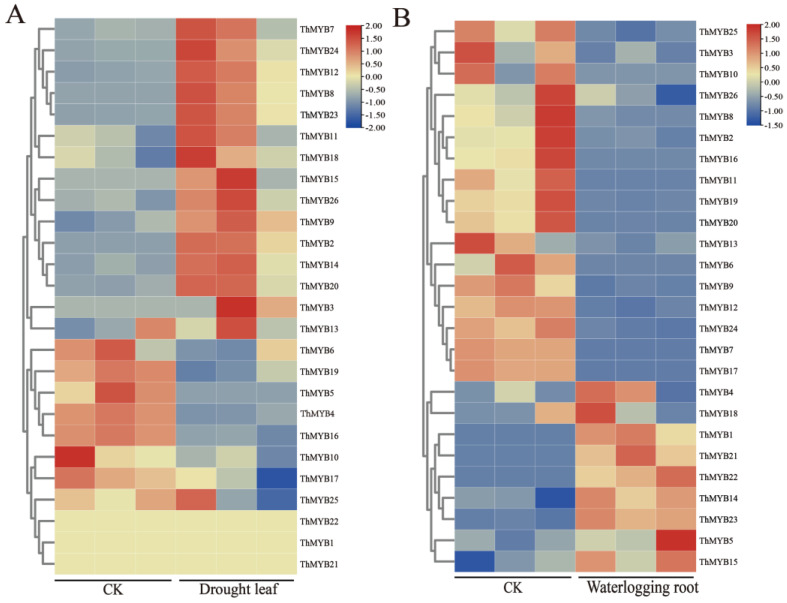
Expression profiles of *R2R3-MYB* genes under water stress. (**A**)The expression pattern of leaves under drought stress. (**B**)The expression pattern of roots under waterlogging stress. The color bar represents the normalized TPM values as follows: red—high expression level; blue—low expression level; and yellow—no expression.

In plants, many *R2R3-MYB* genes are related to abiotic stress [13]. For example, tomato *MYB49* enhanced resistance to water deficit and *FtMYB13* from Tartary buckwheat improved drought tolerance in *Arabidopsis* [41,42].To investigate the regulatory roles of *R2R3-MYB* genes in *T. hemsleyanum* under different water stress, we mapped their expression profiles. As is shown in Figure 3, the results suggest that the *R2R3-MYB* genes showed diverse expression patterns among drought and waterlogging stages. Most *R2R3-MYB* genes displayed higher expression profiles under a drought condition, and a handful of *R2R3-MYB* genes were un-regulated under waterlogging stress as well. In Figure 3A, the genes, *ThMYB7*, *ThMYB24*, *ThMYB12*, *ThMYB8*, *ThMYB23*, *ThMYB18*, *ThMYB26*, *ThMYB15*, *ThMYB9*, *ThMYB2*, *ThMYB14*, *ThMYB20*, and *ThMYB3* were significantly different between the control group and the experimental group, whose expression levels were all increased. Three genes, *ThMYB22*, *ThMYB1*, and *ThMYB21*, showed no expression in the leaves. On the other side, in Figure 3B, the expression of *ThMYB4*, *ThMYB1*, *ThMYB21*, *ThMYB22*, *ThMYB14*, *ThMYB23*, *ThMYB5*, and *ThMYB15* were upregulated. Comparing the expression of *R2R3-MYB* genes in different water treatments, we could find significant differences, suggesting that specific *R2R3-MYB* genes may function on different water stresses. Collectively, these results imply that these *R2R3-MYB genes* responded to water stress in *T. hemsleyanum* and provide further evidence for determining *R2R3-MYB* as potential regulators.

### 3.5. Protein Interaction Networks and Tertiary Structure Prediction

**Figure 4:**
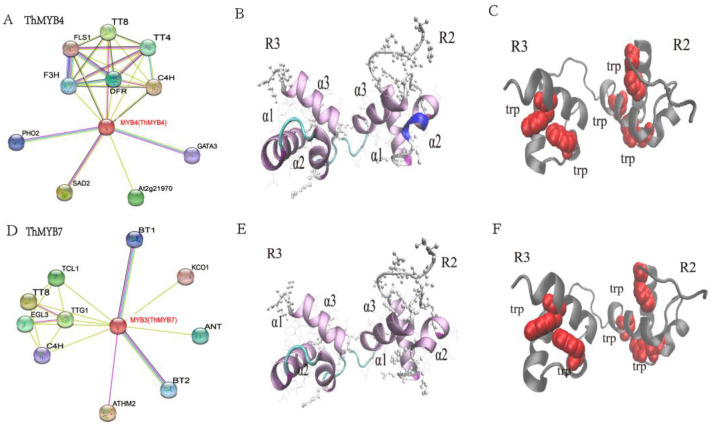
Protein interaction networks and tertiary structure of ThMYB4 and ThMYB7. (**A**,**D**) The protein interaction networks of ThMYB4 and ThMYB7. (**B**,**C**) The tertiary structure of the ThMYB4 protein. (**E**,**F**) The tertiary structure of the ThMYB7 protein.

Based on the R2R3-MYB-associated proteins and protein complexes, it was soon discovered that ThMYB4 and ThMYB7 closely interacted with flavonoid-related proteins, which is also consistent with the evolutionary analysis. In the two protein–protein interaction networks (Figure 4A,D), ThMYB4 and ThMYB7 interacted directly with 20 identified proteins. Among them, the C4H, DFR, F3H, FLS1, and TT4 proteins were involved in flavonoid biosynthesis [43,44]. It can be inferred that ThMYB4 and ThMYB7 interact with the proteins in flavonoid biosynthesis. Moreover, MYB, bHLH and WDR are reported to form protein complexes (MYB-bHLH-WDR) to regulate flavonoid biosynthesis [45]. As shown in in Figure 4A, there was a bHLH (TT8) and not a WDR protein interacting with ThMYB4, indicating that ThMYB4 might function as a MYB-bHLH complex. In addition, two bHLH (TT8 and EGL3) proteins and one WDR protein were found to interact with ThMYB7, suggesting that ThMYB7 might act as a MYB-bHLH-WDR complex.

As a fundamental and crucial component in the study of protein functions, the tertiary structure of the ThMYB4 and ThMYB7 proteins was analyzed. The 3D models were computed using the SWISS-MODEL server homology modeling pipeline [38]. The most apt tertiary structure was chosen based on the statistical potentials of mean force scoring methods. The second and third helices of each repeat build a helix-turn-helix (HTH) structure with three regularly spaced tryptophan (or hydrophobic) residues (Figure 4B,C,E,F), forming a hydrophobic core in the 3D HTH structure [15]. All of these were consistent with the special characterization of R2R3-MYB proteins [46].

### 3.6. Transcriptional Activation Activity and Subcellular Localization of the ThMYB4 and ThMYB7

**Figure 5:**
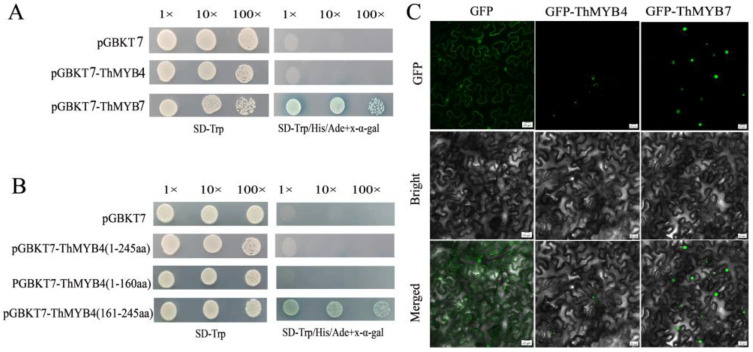
Subcellular location and transcriptional activation activity of *ThMYB4* and *ThMYB7*. (**A**,**B**) Transactivation assays of *ThMYB4* and *ThMYB7* transcription factors. (**C**) The subcellular localizations of *ThMYB4* and *ThMYB7* genes.

We performed subcellular localization and transactivation activity analyses to characterize the properties of *ThMYB4* and *ThMYB7*. Compared to the control, the fluorescence signals of the tobacco cell inserted in the subcellular localization vectors with ThMYB4 and ThMYB7 were observed only in the nucleus, suggesting that ThMYB4 and ThMYB7 were both nuclear localization proteins (Figure 5C). The transactivation abilities of ThMYB4 and ThMYB7 were analyzed using a yeast assay system. The CDS of the two genes were fused to the GAL4 DNA-binding domain, respectively, to generate pGBKT7 plasmids. The plasmids were transformed into the AH109 yeast to assay their abilities to activate the transcription of the GAL4 upstream activation sequence. It was found that pGBKT7-ThMYB7 could grow and show blue colonies on selective media with X-α-gal (Figure 5A). In contrast, pGBKT7 and pGBKT7-ThMYB4 yeast cells displayed growth defects on the SD/-Trp-His-Ade with X-α-gal media (Figure 5A). These results indicate that the complete gene sequences of ThMYB7 did have transactivation abilities, but ThMYB4 did not. To further explore the transactivation ability of ThMYB4, it was divided into N-terminal (1–160 amino acids) and C-terminal (161–245 amino acids) regions and fused into pGBKT7 [47]. As a result, only the C-terminal of ThMYB4 grew on the SD/-Trp-His-Ade media with X-α-gal, meaning that the transactivation activity of *ThMYB4* was relied on its C-terminal (Figure 5B). This phenomenon has also been reported in other literature [47]. However, using a yeast assay system to test the transcriptional activation activity of ThMYB4 and ThMYB7 has some limitations, for there are many other mechanisms for transcriptional activation in eukaryotes. An activation domain can be brought to the DNA by either directly binding to DNA (through its DNA binding domain) or interacting with other DNA-bound proteins [48]. Therefore, the transcriptional activation effects of the ThMYB4 and ThMYB7, especially on the flavonoid metabolic pathways, deserve further study.

### 3.7. Expression of the Key Enzyme Genes in Flavonoid Biosynthesis

**Figure 6:**
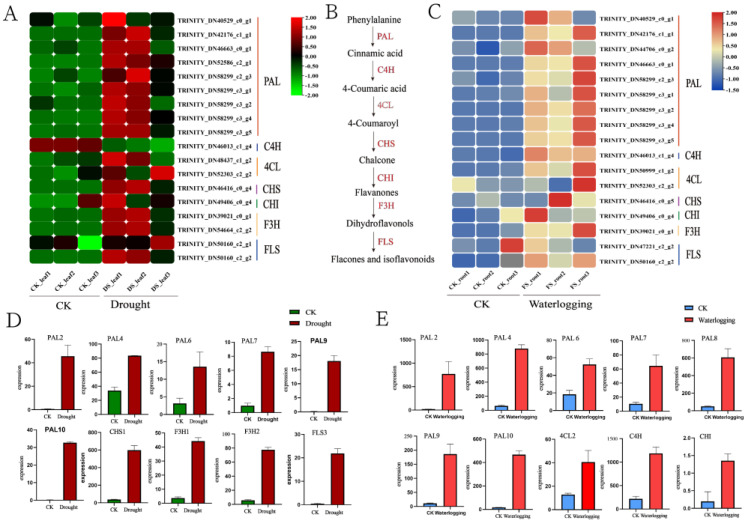
The expression profiles of key enzyme genes. (**A**,**C**) The heatmaps indicate expression levels of flavonoid biosynthetic genes under drought stress and waterlogging stress. (**B**) The flavonoid pathway. PAL—Phenylalanine ammonia-lyase; C4H—cinnamate 4-hydroxylase; 4CL—4-coumarate-CoA ligase; CHS—chalcone synthase; CHI—chalcone isomerase; F3H—flavanone 3-hydroxylase; FLS—flavonol synthase. (**D**,**E**) Expression profiles of the genes involved in flavonoid biosynthesis under water stress.

The flavonoid pathway is a branch pathway of phenylpropane metabolism, and the *PAL*, *C4H*, *4CL*, *CHS*, *CHI*, *F3H*, and *FLS* genes play important roles in flavonoid biosynthesis [49,50]. When plants were subjected to abiotic stress, the expression levels of these key enzyme genes changed [51,52]. This phenomenon also appeared in our results. As shown in Figure 6A,C, the expressions of some key enzyme genes changed after different treatment, and the expression levels of most flavonoid biosynthetic genes were increased under water stress. In Figure 4D, drought stress increased the expression level of six *PAL* genes (*PAL2*, *PAL4*, *PAL6*, *PAL7*, *PAL9*, and *PAL10*), one *CHS* gene (*CHS1*), two *F3H* genes (*F3H1* and *F3H2*), and one *FLS* gene (*FLS3*). In addition, the transcripts of seven *PAL* genes (*PAL2*, *PAL4*, *PAL6*, *PAL7*, *PAL8*, *PAL9*, and *PAL10*), one *4CL* gene (*4CL2*), one *C4H* gene (*C4H*) and one *CHI* gene (*CHI*) exhibited obvious increases compared with the control treatment (Figure 6E). These results indicate that these gene transcripts were mainly induced in the *T. hemsleyanum* after drought or waterlogging treatment and may promote the accumulation of flavonoids.

### 3.8. Correlation Analysis of the R2R3-MYB and Flavonoid Biosynthetic Genes

**Figure 7:**
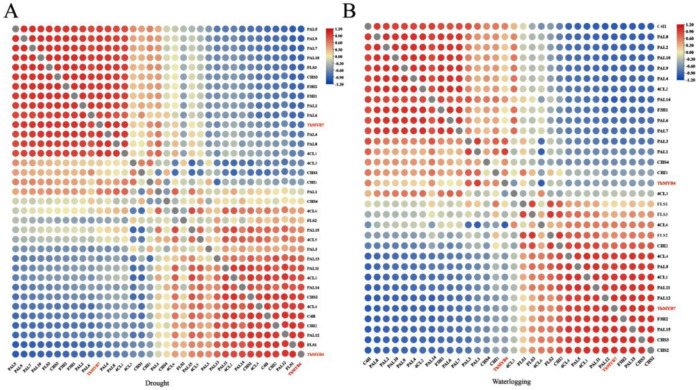
Heatmap of the correlation between *ThMYB4* and *ThMYB7* genes and flavonoid biosynthesis genes under water stress. (**A**) The correlation between *ThMYB4* and *ThMYB7* and flavonoid biosynthesis genes under drought stress. (**B**) The correlation between *ThMYB4* and *ThMYB7* and flavonoid biosynthetic genes under waterlogging stress. The colors represent correlation coefficients: the red color on the key shows positive correlations, with higher positive correlations being closer to positive one (+1), while blue shows negative correlations, with lower correlations being closer to negative one (−1).

Based on the above results, we hypothesized that there may be some connection between *ThMYB4*, *ThMYB7*, and flavonoid biosynthetic genes [53]. To further understand the internal connection, we analyzed the correlation coefficients between *ThMYB4*, *ThMYB7*, and flavonoid biosynthetic genes based on our transcriptomic data. In Figure 7A, under drought stress, *ThMYB4* expression showed a positive correlation with *PAL3*, *PAL11*, *PAL12*, *PAL13*, *PAL14*, *PAL15*, *FLS1*, *CHI2*, *C4H*, *4CL4*, *CHS2*, *4CL2*, *4CL5*, and *4CL6*. *ThMYB7* was positively related to the transcription levels of *PAL1*, *PAL2*, *PAL14*, *PAL5*, *PAL6*, *PAL7*, *PAL8*, *PAL9*, *PAL10*, *FLS3*, *CHI*, *CHS3*, *F3H1*, *F3H2*, and *4CL1.* Additionally, under waterlogging stress, *ThMYB4* had positive correlation with *PAL1*, *PAL3*, *PAL14*, *CHS4*, *C4H*, *CHI1*, and *CHS4*. *ThMYB7* was positively related to the transcription of *PAL5*, *PAL11*, *PAL12*, *PAL15*, *CHS2*, *CHS3*, *F3H2*, *FLS3*, *4CL1*, *4CL4*, *4CL6 CHI2*, and *FLS3.* Consequently, we found that *ThMYB4* and *ThMYB7* were closely correlated with flavonoid biosynthetic genes, laying an important foundation for our subsequent exploration and providing support for us to choose *ThMYB4* and *ThMYB7* as candidate genes.

### 3.9. Identification of Transgenic Hairy Roots and Expression of Flavonoid Biosynthetic Genes

**Figure 8:**
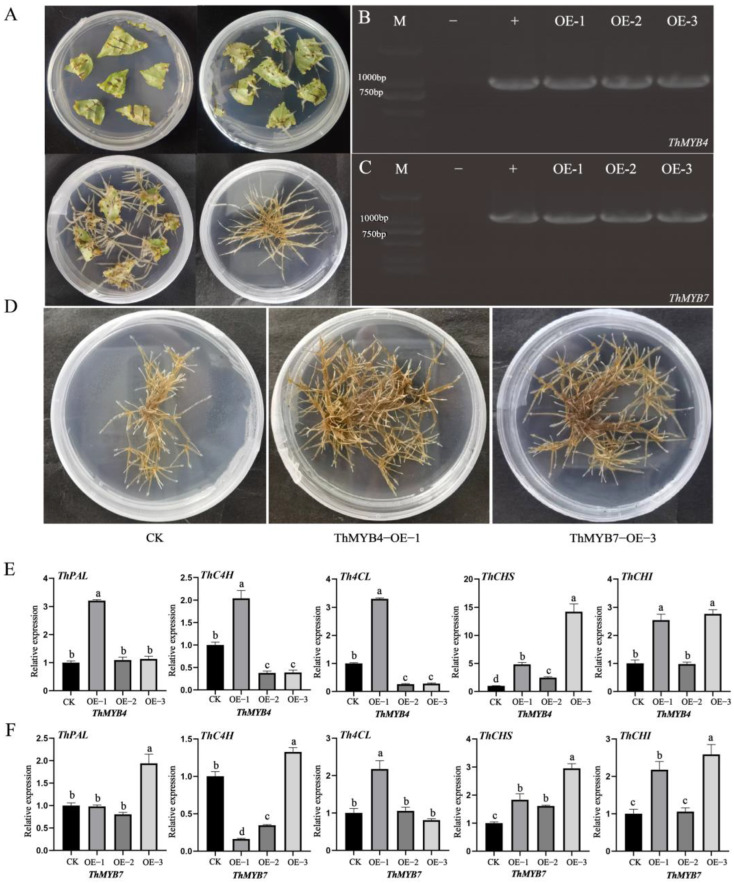
The phenotype and identification of transgenic hairy roots, and the relative expression levels of flavonoid biosynthetic genes in *ThMYB4* and *ThMYB7* transgenic hairy roots. (**A**) The growth process of hairy roots. (**B**,**C**) PCR analysis of *ThMYB4* and *ThMYB7* in independent transgenic hairy roots. M, 2000 maker; −, non-transgenic hairy root lines; +, overexpressed plasmids; OE-1/2/3, the transgenic hairy root lines. (**D**) The changes in color under transgenic hairy roots. (**E**,**F**) The expression *ThPAL*, *ThC4H*, *Th4CL*, *ThCHS*, and *ThCHI* in *ThMYB4*- and *ThMYB7*-overexpressing hairy roots, determined by qRT-PCR amplification. The maximum average was marked by the letter a, the second by the letter b, and so on. The same marked letters represented no significant difference (*p* > 0.05), and different marked letters represented significant difference (*p* < 0.05).

Hairy roots induced by *Agrobacterium rhizogenes* infection have the ability to synthesize secondary metabolites similar to the original plants, and they are also easy to reproduce and grow. Hence, hairy roots are widely used to investigate the regulation of plant secondary metabolism [54]. To verify the regulating effect of *ThMYB4* and *ThMYB7* on flavonoid biosynthesis, the overexpression vectors of *ThMYB4* and *ThMYB7* were constructed to induce transgenic *T. hemsleyanum* hairy roots. The growth process of the hairy roots is displayed in Figure 8A. As shown in Figure 8B,C, the positive transgenic lines of *ThMYB4* and *ThMYB7* were identified by PCR using a specific primer. To determine whether the flavonoid biosynthetic genes were regulated by *ThMYB4* and *ThMYB7* in *T. hemsleyanum*, the expression of the five flavonoid biosynthetic genes (*ThPAL*, *ThC4H*, *Th4CL*, *ThCHS*, and *ThCHI*) in transgenic hairy roots were analyzed by qRT-PCR. Compared with the control, the expression of *ThPAL*, *ThCHS*, and *ThCHI* genes was increased in the *ThMYB4*-overexpressing hairy roots (Figure 8E). In Figure 8F, *ThPAL*, *Th4CL*, *ThCHS*, and *ThCHI* genes were upregulated in the *ThMYB7*-overexpressing hairy roots compared with the control. Notably, the *ThCHS* and *ThCHI* gene expression levels were of significant difference. These results suggest that *ThMYB4* and *ThMYB7* were important regulators for *ThCHS* and *ThCHI*. As the central precursor of flavonoids, chalcone was synthesized by tyrosine and phenylalanine via the sequential reaction of PAL, C4H, 4CL, and CHS, and then converted into the corresponding flavanones by the action of CHI [55]. Therefore, the increased expression of the *ThCHS* and *ThCHI* genes in *ThMYB4* and *ThMYB7*-overexpressing hairy roots may result in the promotion of flavonoid accumulation. As displayed in Figure 8D, the color of ThMYB4-OE-1 and ThMYB4-OE-3 hairy root lines with significant increases in the flavonoid biosynthetic genes were darker than the color in the non-transgenic hairy roots, suggesting more flavonoids were accumulated in both the *ThMYB4* and *ThMYB7* transgenic hairy roots. Therefore, it can be confirmed that both *ThMYB4* and *ThMYB7* could positively regulate the flavonoid biosynthesis, and it was consistent with *McMYB4* but opposite to *AtMYB4* in flavonoid biosynthesis regulation. This is instructive for the regulating the flavonoid accumulation in *T. hemsleyanum*. This will shed new light on the promotion of flavonoid production and the medicinal value of *T. hemsleyanum* by manipulating transcription factors.

## 4. Conclusions

In this study, the *ThR2R3-MYB* genes were identified and phylogenetically divided into 15 subfamilies. Through the integration of gene expression patterns, correlation analysis, phylogenetic trees, and protein interaction works, the *ThMYB4* and *ThMYB7* genes were determined to most likely be involved in the accumulation of flavonoids. Notably, the expression levels of the flavonoid biosynthetic genes were increased in *ThMYB4-* and *ThMYB7*-overexpressing transgenic hairy roots. Taken together, our study generates an important resource that can be used for further studies of *ThR2R3-MYB* genes in *T. hemsleyanum* and contributes valuable information to investigate the functions of *ThR2R3-MYB* on flavonoid accumulation under water stress.

## Figures and Tables

**Table 1:** Physicochemical properties and subcellular location prediction of R2R3-MYB proteins.

Gene Name		Molecular Weight	pI	Instability Index	Aliphatic Index	GRAVY	Subcellular Localization
*ThMYB1*		85,902.32	8.90	56.33	77.72	−0.793	Nucleus
*ThMYB2*	*1*	29,951.35	6.66	46.42	72.83	−0.556	Nucleus
*ThMYB3*		13,880.10	10.03	57.11	80.25	−0.822	Nucleus
*ThMYB4*	*2*	27,664.39	8.60	54.75	68.85	−0.745	Nucleus
*ThMYB5*	*4*	18,984.85	9.69	39.07	81.23	−0.787	Nucleus
*ThMYB6*		13,220.45	9.99	49.39	81.23	−0.742	Nucleus
*ThMYB7*		31,423.51	9.10	57.18	76.52	−0.78	Nucleus
*ThMYB8*		28,842.09	6.07	45.36	66.43	−0.578	Nucleus
*ThMYB9*		28,112.56	8.57	62.37	70.75	−0.651	Nucleus
*ThMYB10*		24,574.13	9.76	62.24	73.02	−0.842	Cytosol
*ThMYB11*		36,403.92	6.19	40.10	73.39	−0.609	Nucleus
*ThMYB12*		38,682.27	6.09	63.79	71.98	−0.644	Nucleus
*ThMYB13*		26,657.84	8.25	70.53	59.74	−0.967	Nucleus
*ThMYB14*		29,822.45	6.41	61.75	66.09	−0.688	Nucleus
*ThMYB15*		21,199.05	5.19	46.10	54.38	−1.006	Nucleus
*ThMYB16*		16,522.94	9.79	54.03	69.78	−1.223	Nucleus
*ThMYB17*		34,909.27	8.69	42.89	63.84	−0.692	Nucleus
*ThMYB18*		37,590.73	10.56	62.63	72.38	−0.625	Nucleus
*ThMYB19*		32,778.41	6.81	48.68	61.88	−0.533	Nucleus
*ThMYB20*		51,441.56	5.83	53.82	72.93	−0.653	Nucleus
*ThMYB21*		29,780.29	8.28	60.91	67.29	−0.614	Nucleus
*ThMYB22*		13,054.74	9.80	58.47	66.67	−0.441	Cytosol
*ThMYB23*		33,086.11	8.36	40.5	69.02	−0.43	Nucleus
*ThMYB24*		14,153.01	5.21	57.43	76.80	−0.578	Nucleus
*ThMYB25*		17,449.79	6.75	53.18	66.86	−0.758	Nucleus
*ThMYB26*		34,271.40	5.28	49.20	73.63	−0.494	Nucleus

## Data Availability

The data and materials that support the findings of this study are available from the corresponding authors upon reasonable request.

## Supplementary Materials

The following supporting information can be downloaded at: https://www.mdpi.com/article/10.3390/biom13030531/s1, Table S1: The primers of the qRT-PCR and transgenic hairy root identification.
